# Reimplantation of Thymus at the First Superior Cavo-Pulmonary Anastomosis and Its Outcome

**DOI:** 10.7759/cureus.76599

**Published:** 2024-12-29

**Authors:** Sachin Talwar, Vuribindi M Reddy, Amitabh Satsangi, Vishal V Bhende, Sherrin Jacob, Aruna Nambirajan, Akshya K Bisoi

**Affiliations:** 1 Cardiothoracic and Vascular Surgery, All India Institute of Medical Sciences, New Delhi, IND; 2 Pediatric Cardiac Surgery, Bhanubhai and Madhuben Patel Cardiac Centre, Shree Krishna Hospital, Bhaikaka University, Karamsad, IND; 3 Pathology, All India Institute of Medical Sciences, New Delhi, IND

**Keywords:** fontan completion, immunity, living tissue, reoperation, thymus

## Abstract

During bidirectional cavo-pulmonary anastomosis (bidirectional Glenn; BDG), the thymic tissue is often excised to facilitate the exposure of the superior vena cava and its junction with the innominate vein. Subsequently, it is discarded. Since the last two decades, the lead author (ST) has pursued anchoring the excised thymus in its position by suturing it to the opposite unexcised thymic lobe. At the time of completion Fontan, the authors found the excised thymic tissue to be living on electron microscopy. In this report, the authors describe one such patient. The advantages of this approach have been discussed here. The techniques of performing the BDG and the Fontan are not discussed.

## Introduction

In patients with an anatomically or functionally univentricular heart, the Fontan operation is the final desired palliation. In the current era, this operation is commonly staged after a prior bidirectional Glenn (BDG) operation that has been performed earlier in infancy/early childhood. At the time of performing the BDG, the ipsilateral lobe of the thymus on the side of the superior vena cava (SVC) is often removed to facilitate the exposure of the SVC and the SVC-innominate vein (IV) junction. The opposite lobe of the thymus is left intact. At the end of the procedure, the resected thymic lobe is discarded presuming that it has lost its vascularity. However, over the last two decades, the lead author (ST) has persistently anchored the excised thymic tissue in its normal anatomic position by suturing it to the opposite lobe of the thymus. In this report, the detailed histologic findings in the same specimen of thymus at the time of the final Fontan palliation are presented. The advantages of this strategy are discussed. The literature search, conducted across multiple medical databases, revealed no reports or studies on the reimplantation of thymic tissue. Due to the absence of available data, it is challenging to comment on the potential risks or limitations such as infection or inflammation. However, based on the clinical experience of the lead author (ST) spanning more than two decades, no adverse effects, including infection or inflammation, have been observed. This safety record has reinforced confidence in the approach and supports its continued application. Nonetheless, we acknowledge that further studies with larger cohorts and long-term follow-up would be valuable to confirm the generalizability and safety of this novel strategy.

## Case presentation

The Institutional Ethics Committee, All India Institute of Medical Sciences, New Delhi approved the publication of the findings in this patient vide approval no. AIIMS A1453/07.06.2024 dated 11.06.2024. The patient's identity is not disclosed.

An 8.7-year-old patient with a diagnosis of tricuspid atresia, ventricular septal defect, atrial septal defect, and severe pulmonary stenosis presented to us for the completion Fontan operation. He had undergone the BDG operation at eight months of age. At BDG the excised thymic tissue was not discarded. After the completion of the BDG, the pericardium was completely closed. Subsequent to this, the excised right lobe of the tissue was sutured to the left lobe of the thymus using interrupted sutures.

At the time of the completion Fontan, some living tissue, presumably the thymic tissue, was identified in the position of the right lobe of the thymus. This tissue completely covered the aorta and the SVC. After removing it, the tissue had grossly normal architecture compared to that of the thymus of a normal patient. It was not apparent that the thymus had previously been removed. This favored minimal adhesions over the great vessels and easy dissection during reoperation.

This tissue was excised and was sent for histopathological examination. The Fontan operation was completed as usual and was uneventful.

Gross specimen details

A gross specimen measuring 5 x 3 x 0.2 cm was sent for histopathological examination. It was yellowish brown in color and soft in consistency. It was identified as morphologically similar to thymus.

Light microscopy findings

On light microscopy with hematoxylin and eosin stain, this tissue parenchyma was noted to be arranged in lobules. These lobules were of varying sizes separated by edematous connective tissue. The cortex showed densely packed lymphoid cells, i.e. thymocytes with few interspersed epithelial cells with moderate to abundant cytoplasm.

Electron microscopy findings

**Figure 1:**
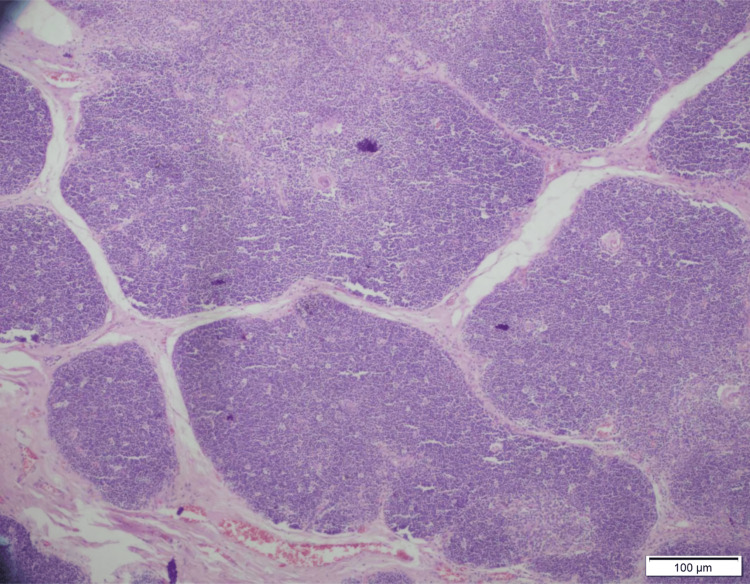
Light microscopy with hematoxylin and eosin stain (4× magnification) showing the lobulated architecture of normal thymic parenchyma. Each lobular compartment is composed of cortex (darker zone) and medulla (paler zone). Scale bar: 100 μm. Image Credits: Dr. Sherrin Jacob/Dr. Aruna Nambirajan.

**Figure 2:**
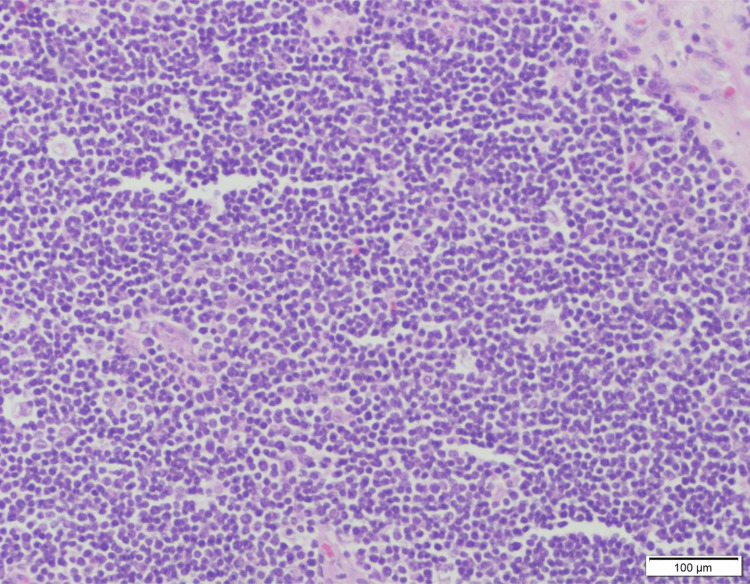
Light microscopy with hematoxylin and eosin stain (20× magnification) showing thymocytes predominantly with few interspersed epithelial cells. Scale bar: 100 μm. Image Credits: Dr. Sherrin Jacob/Dr. Aruna Nambirajan.

**Figure 3:**
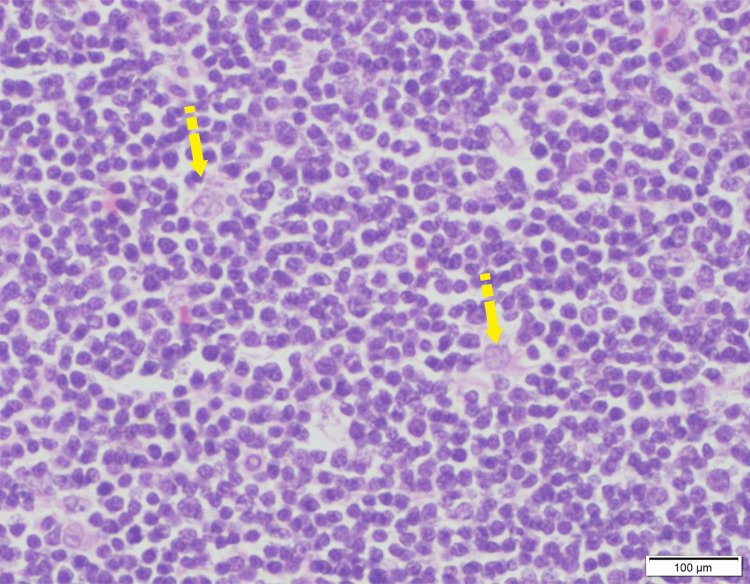
Light microscopy with hematoxylin and eosin stain (40× magnification) showing sheets of cortical thymocytes in different grades of differentiation. The yellow arrows point to the scattered thymic epithelial cells identified by the vesicular nucleus and increased cytoplasm. Scale bar: 100 μm. Image Credits: Dr. Sherrin Jacob/Dr. Aruna Nambirajan.

**Figure 4:**
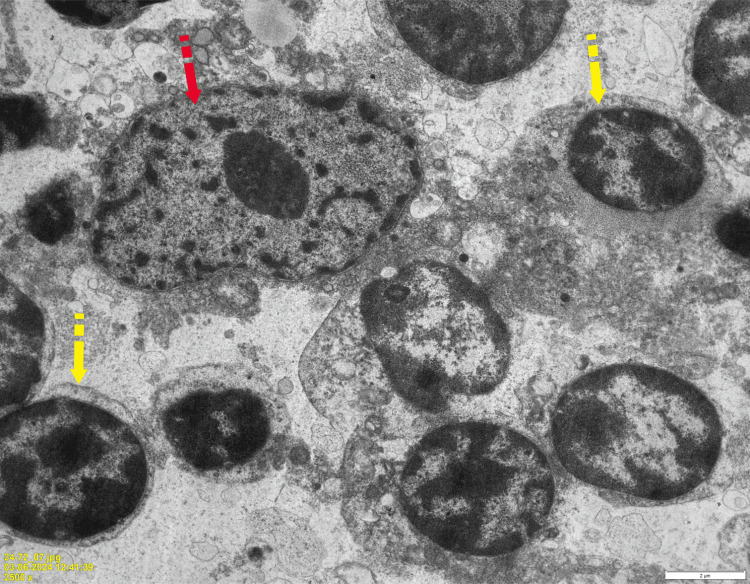
Electron microscopy image demonstrating thymocytes with interspersed epithelial cells. Thymic epithelial cell (red arrow) surrounded by thymocytes (yellow arrow). Scale bar: 2 μm. Image Credits: Dr. Sherrin Jacob/Dr. Aruna Nambirajan.

**Figure 5:**
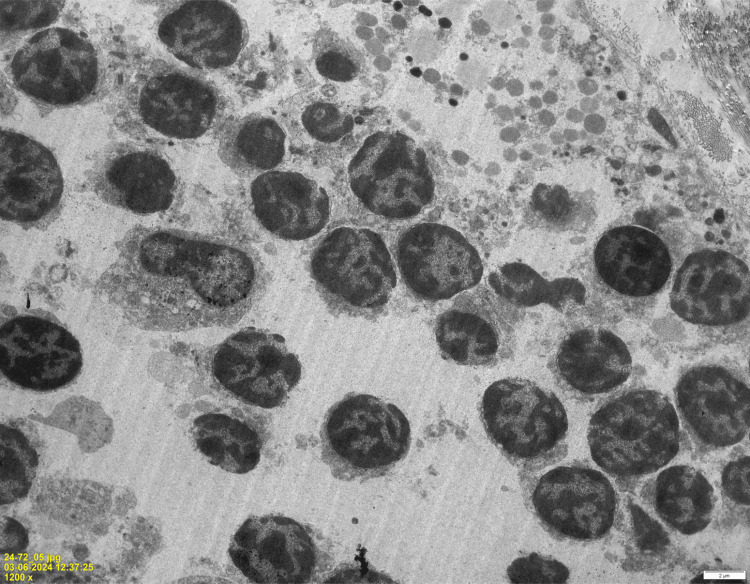
Electron microscopy image demonstrating thymocytes in different stages of differentiation. Scale bar: 2 μm. Image Credits: Dr. Sherrin Jacob/Dr. Aruna Nambirajan.

On electron microscopy, thymus tissue was identified with preserved architecture. Thymocytes in various stages of maturation were noted with interspersed epithelial cells. The detailed findings of pathological specimens are presented in Figures 1-5.

## Discussion

Revascularization and viability of tissue that is excised and reimplanted in a few hours in the same person is known. We used that as a structural advantage in our patient for a safe reoperation. During the Fontan completion following a BDG procedure, earlier in life, we could identify both the lobes of the thymus as in any normal individual. The histopathologic description and the advantage of this strategy are listed in this report.

There is considerable uncertainty over the thymus's true significance in both adults and children. In patients undergoing surgery for congenital heart defects, the bulky thymus is removed in total or in parts to better visualize the cardiac structures [1]. The excised thymic tissue is commonly discarded. However, as discussed above, we have refrained from this policy because of the following: (a) the thymus partially covers the heart and the major vessels and is located in the upper anterior mediastinum, posterior to the sternum; thus, it protects the cardiac structures during reoperation [1]. (b) By offering a microenvironment where bone marrow progenitor cells multiply and grow into mature T-cells, the thymus plays a critical role in the development of T-cells [2].

T-cell development in vertebrates relies on a functional thymus, which varies throughout life. The thymus is nearly fully grown at birth and attains its peak functionality during adolescence. Subsequently, the total volume begins to diminish while gradually converting active tissue into adipose tissue. This phenomenon is referred to as thymic involution. Despite the loss in weight, size, and activity, the thymus continues to function as the primary site for T-cell development and maturation into adulthood [3].

Cardiothoracic surgery and concurrent thymectomy conducted at a younger age facilitate quick and substantial alterations in the blood profile. Numerous research has indicated that thymectomy induces immunological changes, characterized by diminished CD4+ and CD8+ T-cell counts, decreased proportions of "recent thymic emigrants," and a reduced quantity of naive T-cells [4]. In contrast to earlier investigations, the research conducted by Van Gent et al. demonstrated that the T-cell pool returns to normal after five years. The gradual recuperation of thymic tissue, as evidenced by magnetic resonance imaging (MRI) in the follow-up, may elucidate this trend [5]. In our patient, we have often wanted to use the benefit of the thymus at the anatomical location for a safer reoperation during the Fontan procedure. The fact that the thymus remains viable allows it to grow further with age and prevents adhesions favoring dissection. However, there might be a minor benefit in maintaining the immunity in infants after a primary operation. In our patient, the excised thymus specimen that was sent for routine and electron microscopy was found to be viable and was completely indistinguishable from a normal thymus. We preferred electron microscopy to structurally demonstrate the living nature of the tissue, rather than antibody testing for thymocytes, which can be considered in further cases as feasible.

Despite the extensive variation in follow-up, the existing long-term trials did not reveal clinically significant alterations. Preserving thymic tissue is frequently advised [5]. Although this recommendation appears to lack scientific foundation, we might be able to provide supporting evidence in preserving the thymus at primary procedures in the future using antibody testing that may be more specific in demonstrating this.

## Conclusions

We recommend that suturing the excised tissue to the unexcised opposite lobe of the thymus at the first BDG should be considered. This approach protects the great vessels, making the reoperation safer. In addition, it may preserve the immune status.
